# Supplementary material for: Toxic Effects of Bisphenol AF Exposure on the Reproduction and Liver of Female Marine Medaka (Oryzias melastigma)
Source: Animals (Basel). 2024 Jan 10;14(2):222. doi: 10.3390/ani14020222 (PMC10812438; doi:10.3390/ani14020222)
Supplement: Supplementary file 1 [file animals-14-00222-s001.zip › animals-2766444-supplementary.pdf]

Table S1. Fluorescent quantitative PCR primers for marine medaka

Primer name	primer sequence	Genebank ID	Product size (bp)
vtgr	F:GACAACCCGGTCTACCTGAA;<br>R:CTGGATTGAAGGAGGGATGA	XM_024262816.2	164
vtg1	F:TTGGCAGAGATGCAGCAGCGGT;<br>R:GGAAATGCAGGACACCCCAGTAGCC	XM_024279512.2	83
vtg2	F:AAGCCCTCAACCACTGGCTCCCTAAT;<br>R:TGGGCAGCGCCGTTCAAGATGTTGAT	XM_024279510.2	93
star	F:AGAAGGCTATCAGCATCCTCAGTG;<br>R:AGGCAGTACCTTACTCAGGACCTT	XM_024276393.2	93
lhr	F:CCTGGTGGTGTGCTACTGCTAC;<br>R:CGGCGGAGATGGCGAAGAAG	XM_024297983.2	162
fshr	F:GCGTGTGCGGCTGCTACC;<br>R:CGAGATGGCGAAGAAGGAGATGG	XM_024285506.2	153
erβ	F:TGATCCAGGAGGCTGAGCTCCACGA;<br>R:ACCGCTGACGGAGGCTGTTGTGAT	XM_024291918.2	121
cyp19a	F:ACCTCGCGTTTTGGCAGCAAACA;<br>R:TTTCCACAGCGCCACGTTGTTGT	XM_024296015.2	91
cyp17a1	F:CAGACTACAGCGACCACGTT;<br>R:CTGGATAATGGATCAGGTAGGT	XM_024296725.1	220
cyp11b	F:GCATTGGCCTGTTCTCCTCATCT;<br>R:CGGAGGGGTGGTTGTTAGCATT	XM_024267121.2	87
cyp11a	F;CGTTTGGGTTTACTGCTGGACTAC;<br>R:GCGGTCAGCTTGGTTGAATATCC	XM_024282518.2	187
erα	F:TCGCCGCTGTTGTGCTGTGATGTT;<br>R:TCCTGGATCTGAGTGCGGGTCCGA	JF907629	84
arα	F:TTTGATGAACTGCGGACCTCCTAC;<br>R:AACTGGTGCAATTTCCTCACAACC	XM_024283708.2	144
3βhsd	F:TGGTCAACCCCGTCTATGTG;<br>R:GGTGTCGTCAGTGGCGAAGT	XM_024260561.2	123
17βhsd	F:CGCTACCTCCACAAAGTTGTTGTC;<br>R;AGTTCTGCCTCAACAGTTTCACCT	XM_024271605.2	138
chgh	F:ATGCCGACTATCCTGTGACC;<br>R:AGCGTCCAAGAGTCAGAACC	XM_024282618.2	103
chgl	F:CAAAGTTCGTGTCTCGACCA;<br>R:TCAGCATCAAGGGGATAAGC	XM_024281848.1	140
fasn	F:TCTCCTTACTGAACTGTGTAGCA;<br>R:CCATTCCTGAGCAGACATACCA	XM_024261534.2	148
dgat2	F:CACTGTGCTGCTGATCTACATT;<br>R:GAGGATCTCCTTCCACCTTGTT	XM_024266114.2	115
apoba	F:GGCTTCTTCTCTGAATCCATCTC;	XM_024266388.2	126

	R:GTCCTCTGTCACCTGTCTCTTC		
apocl	F:GATGCGACCTACACTAAGATAAGA; R:ATGTTGAAGTGAACCAGTTCCT	XM_024267688.2	137
cyp7a1	F:GTCTTCAAGAGTGCCTACAGTG; R:AAGGTGGATAAGGAGTCGTTCA	XM_024268797.1	132
lpl	F:ACTGAGTGGATGACGGATTACA; R:GTCTGAGCTTCTGCGTTGAATT	XM_024278730.2	144
thf	F:CTTCGTGGTTCTGGTTCT; R:GTTGTCCGCTAGTCTGAT	XM_024260306.2	171
il-8	F:TGCACTGCCGCTGCATCTTGACAG; R:TCTGGGTTCAGGCAAACCTCCTGGC	XM_024289379.2	149
sod	F:TGTACCAGTGCGGGGCCTCACTTCA; R:TGCGGTCACATTTCCCAGGTCCCCA	XM_024266073.1	100
gpx	F:GTGTGCAGAAACGACGTGGCCTGGA; R:TCGCCTTCGATGTCGCTGGTGAGGA	XM_024270129.2	111
cat	F:GCCAACTACCTGCAGATCCCCGTCA; R:AGTTTGGAGCGCCGCCTTGGTTGT	XM_024265602.1	110
cox-1	F:AGTTCGACCCCACGCTGCTGTTCA; R:AAGCTGTCGGGCATCAAAGGGTGC	XM_024299369.2	83
cox-2	F:CAGTGCTGACCGAGCATGGCATCA; R:TTACGACCACCAGCAACCCGTCCT	XM_024282897.2	129
pparα	F:AACAAAGATGGCCTCCTGGTGGCT; R:ATGGAGTTGAAGCGTGTGGCGAAC	XM_024293542.2	147
pparβ	F:TGCATTCCAGGGTTTGTGGACCTCT; R:CTTGTGACAAAGCCTTTACCGTTGGCT	XM_024293543.2	147
rps4x	F:TGCCACCAGGCTCTCCAACAT; R:TTGTCCCTCTCCTCTGCGATGG	XM_024287485.2	109
actb2	F:GCAGGTCATCACCATCGGCAAT; R:CCTCCAGACAGCACAGTGTTGG	XM_024272788.2	172

Table S2. The actual concentration of BPAF in water

Samples	Actual concentration at t_0 (SD) (μg/L)	Actual concentration at t_{48}(SD) (μg/L)	Mean (SD) (μg/L)
Control	n.d	n.d	n.d
BPAF	199.15 (4.94)	177.51 (8.44)	188.33 (12.84)

The symbol SD stands for standard deviation. The recovery efficiency of BPAF was 84%, The detection limit of BPAF is 0.5μg/L.

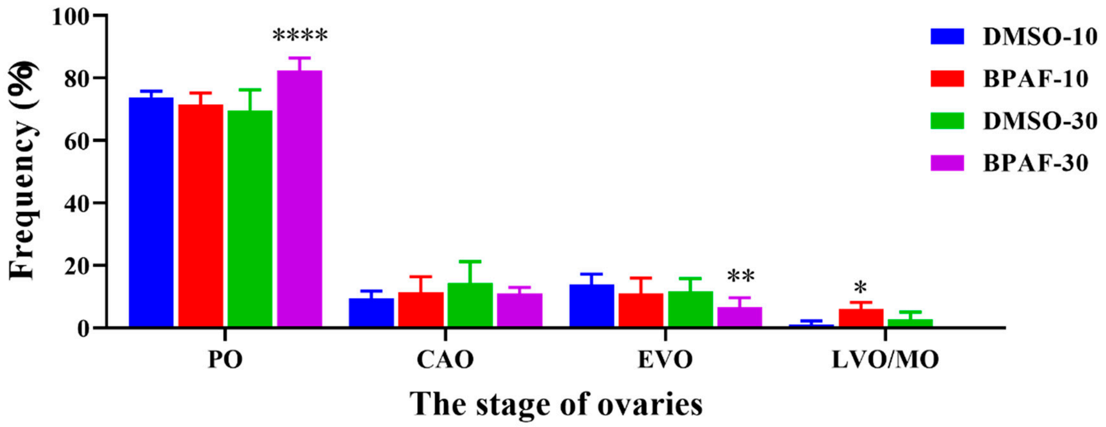

Figure S1 The alterations in relative percentages of each stage of ovaries.

Note: PO: perinucleolar oocytes; CAO: cortical alveolar oocytes; EVO: early vitellogenic oocytes; LVO/MO: late vitellogenic oocytes/mature oocytes; The results are represented as mean ± S.D. (n = 8). Compared to the control (DMSO-10d), statistically significant differences are shown with asterisks (*P ≤ 0.05, **P ≤ 0.01, and ****P ≤ 0.0001).
